# Renoprotective effect of long acting thioredoxin by modulating oxidative stress and macrophage migration inhibitory factor against rhabdomyolysis-associated acute kidney injury

**DOI:** 10.1038/srep14471

**Published:** 2015-09-28

**Authors:** Kento Nishida, Hiroshi Watanabe, Shigeru Ogaki, Azusa Kodama, Ryota Tanaka, Tadashi Imafuku, Yu Ishima, Victor Tuan Giam Chuang, Masao Toyoda, Masumi Kondoh, Qiong Wu, Masafumi Fukagawa, Masaki Otagiri, Toru Maruyama

**Affiliations:** 1 Department of Biopharmaceutics, Graduate School of Pharmaceutical Sciences, Kumamoto University, 5-1, Oe-honmachi, Chuo-ku, Kumamoto 862-0973, Japan; 2 Center for Clinical Pharmaceutical Sciences, School of Pharmacy, Kumamoto University, 5-1, Oe-honmachi, Chuo-ku, Kumamoto 862-0973, Japan; 3 School of Pharmacy, Faculty of Health Sciences, Curtin Health Innovation Research Institute, Curtin University, GPO Box U1987, Perth 6845, WA, Australia; 4 Division of Nephrology, Endocrinology and Metabolism, Tokai University School of Medicine, Isehara 259-1193, Japan; 5 Faculty of Pharmaceutical Sciences, Sojo University, 4-22-1, Ikeda, Nishi-ku, Kumamoto 860-0082, Japan; 6 DDS Research Institute, Sojo University, 4-22-1, Ikeda, Nishi-ku, Kumamoto 860-0082, Japan

## Abstract

Rhabdomyolysis-associated acute kidney injury (AKI) is a serious life-threatening condition. As such, more effective strategies are needed for its prevention. Thioredoxin-1 (Trx), a redox-active and macrophage migration inhibitory factor (MIF) modulating protein, has a short retention time in the blood. We examined the renoprotective effect of long acting Trx that was genetically fused with human serum albumin (HSA-Trx) against glycerol-induced AKI. An intravenous HSA-Trx pre-treatment attenuated the glycerol-induced decline in renal function, compared to a PBS, HSA or Trx alone. HSA-Trx caused a reduction in the tubular injuries and in the number of apoptosis-positive tubular cells. Renal superoxide, 8-hydroxy deoxyguanosine, nitrotyrosine and the plasma Cys34-cysteinylated albumin were clearly suppressed by the HSA-Trx treatment. Prior to decreasing TNF-α and IL-6, HSA-Trx suppressed an increase of plasma MIF level. In LLC-PK1 cells, HSA-Trx decreased the level of reactive oxygen species and lactate dehydrogenase release induced by myoglobin. HSA-Trx treatment resulted in a threefold increase in the survival of lethal glycerol-treated mice. The post-administration of HSA-Trx at 1 and 3 hr after glycerol injection exerted a significant renoprotective effect. These results suggest HSA-Trx has potential for use in the treatment of rhabdomyolysis-associated AKI *via* its extended effects of modulating oxidative stress and MIF.

Rhabdomyolysis is a clinical condition initiated by an acute disruption of skeletal muscle due to physical or chemical damage from a crushing injury, exhaustive exercise, surgery, certain types of medications or toxins^1^. When skeletal muscle is damaged, myoglobin and other intracellular proteins leak into the circulatory system, resulting in myoglobinuria (tea-colored urine), and if this is sufficiently severe it can result in acute kidney injury (AKI). In fact, about 10–50% of patients with rhabdomyolysis develop AKI^2^, representing about 7 to 10% of all such cases^3^. Although interventions have improved, the mortality rate remains as high as 8%^1^,^3^,^4^. Therefore, it would be desirable to develop a therapeutic strategy for both preventing and treating rhabdomyolysis-associated AKI.

The experimental animal model for rhabdomyolysis is produced by disrupting skeletal muscle by a glycerol treatment and is used in studies of the clinical syndrome as well as the mechanisms of AKI^5^. Although the pathogenesis of glycerol-induced AKI is complex and not well understood^6^, it has been proposed that oxidative stress is involved^7^. When myoglobin is released from cells, it becomes concentrated along the renal tubules where it leads to the uncontrolled leakage of reactive oxygen species (ROS), which can cause tubular injury^8^. It has been suggested that heme released from myoglobin and heme proteins such as cytochrome P450 in the kidney functions as critical mediator of such tubular damage^8^,^9^. In addition, Reeder B *et al.* reported that myoglobin itself can exhibit peroxidase-like enzymatic activity^10^.

On the other hand, inflammation also appears to play a role in rhabdomyolysis-associated AKI^11^. In particular, an increase in the number of macrophages^11^ and released inflammatory cytokines such as tumor necrosis factor-α (TNF-α) are typical features in glycerol-induced AKI^12^. In addition, Homsi E *et al.* reported that apoptosis also participates in important pathogenic mechanisms in glycerol-induced AKI^7^,^13^. Therefore, to prevent or treat rhabdomyolysis-associated AKI, the therapeutics would be required to possess anti-oxidative, anti-inflammatory and anti-apoptotic activities with respect to the kidney, especially, in tubular cells.

Thioredoxin-1 (Trx) is a ubiquitous, redox-active, low-molecular-weight protein that has anti-oxidative, anti-inflammatory and anti-apoptotic properties in addition to modulating the macrophage migration inhibitory factor (MIF). Recently, MIF has attracted attention as an exacerbation factor for inflammatory diseases because it promotes the production of TNF-α, interleukin-6 (IL-6) and other cytokines, in addition to ROS^14^,^15^. Although Trx has great potential for use as a therapeutic agent against several types of oxidative stress-related diseases^15^, its short half-life (1 h in the mouse, 2 h in the rat) limits its clinical application. We recently engineered a genetic fusion protein that is comprised of human serum albumin (HSA) and Trx (HSA-Trx) and is expressed by a *Pichia* expression system^16^,^17^,^18^,^19^,^20^,^21^. The pharmacokinetic properties of the HSA-Trx fusion protein are similar to that of HSA itself, and its plasma half-life in mice and rats is 10 times longer than Trx itself^16^,^17^,^18^. Interestingly, HSA-Trx showed a higher distribution in the lungs and kidney than to other organs^16^. HSA-Trx has proven to be therapeutically effective in a septic shock mouse model^16^, as well as in alleviating oxidative stress-related diseases^17^,^18^,^21^. We also found that HSA-Trx exhibited an anti-inflammatory action *via* the inhibition of MIF, consequently suppressing the production of inflammatory cytokines. Furthermore, our recent study demonstrated that HSA-Trx prevents experimental contrast-induced nephropathy and cisplatin nephrotoxicity due to its prolonged anti-oxidative action not only in the extracellular compartment but also inside tubular cells^19^,^20^. It would therefore be expected that HSA-Trx would ameliorate rhabdomyolysis-associated AKI because of its unique long lasting biological activity.

The objective of this study was to investigate the therapeutic impact of HSA-Trx on a mouse model of glycerol-induced AKI. The renoprotective effect, anti-oxidative, anti-inflammative and anti-apoptotic action in addition to the modulation of MIF of HSA-Trx on the model were evaluated *in vivo*. The *in vitro* myoglobin-induced ROS scavenging activity of the fusion protein was also examined using porcine kidney epithelial cells (LLC-PK1 cells). In addition, cell viability assay (lactate dehydrogenase (LDH) release) was also performed. The findings presented herein provide evidence to show that HSA-Trx has the potential for use in the treatment of rhabdomyolysis-associated AKI by virtue of its ability to modulate the effects of oxidative stress and MIF.

## Results

### Effect of HSA-Trx on glycerol-induced renal dysfuncion

The mouse model of rhabdomyolysis-associated AKI, induced by the administration of 10 mL/kg of a 50% glycerol solution, exhibited elevations in blood urea nitrogen (BUN) and serum creatinine (SCr) in time dependent manner for 24 h after the glycerol administration. In addition, decreased in creatinine crearance (CCr) was observed after the glycerol administration, as reported previously (Supplemental Figure 1A–C)^22^,^23^. Plasma oxidative stress markers such as heme, malondialdehyde and hydroperoxide levels reached a maximum at 1 h, and proinflammatory cytokine TNF-α levels were increased at 24 h after the glycerol administration (Supplemental Figure 2A–D). These data indicated the validity of this glycerol-induced AKI model for evaluating the renoprotective effect of HSA-Trx.

**Figure 1:**
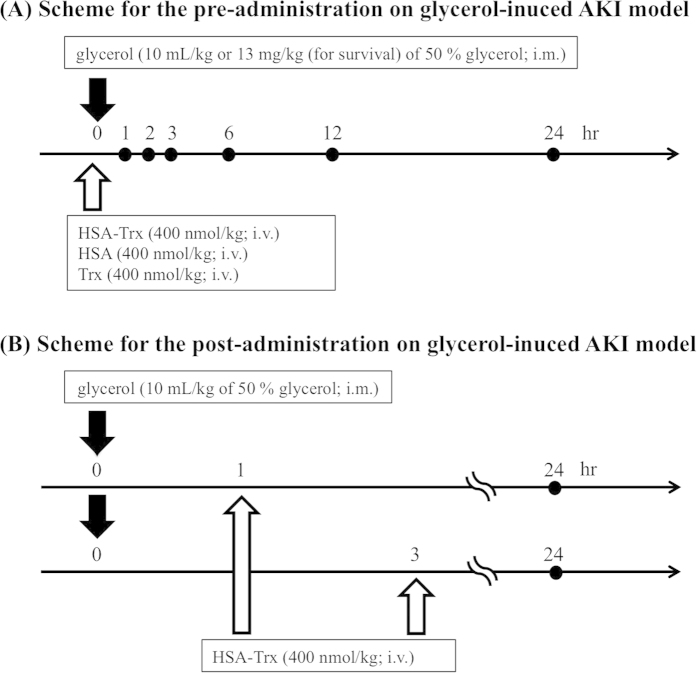
Experimental protocol for the glycerol-induced AKI model. (**A**) HSA-Trx (400 nmol/kg), HSA (400 nmol/kg) or Trx (400 nmol/kg) was administered intravenously immediately prior to the injection of glycerol. The mouse was injected with 10 mL/kg or 13 mL (for survival) of 50% glycerol intramuscularly *via* the two hind-limbs. (**B**) For the post-administration experiment, HSA-Trx (400 nmol/kg) was administered intravenously at 1 hr or 3 hr after glycerol injection. The mouse was injected with 10 mL/kg of 50% glycerol intramuscularly to the two hind-limbs.

**Figure 2:**
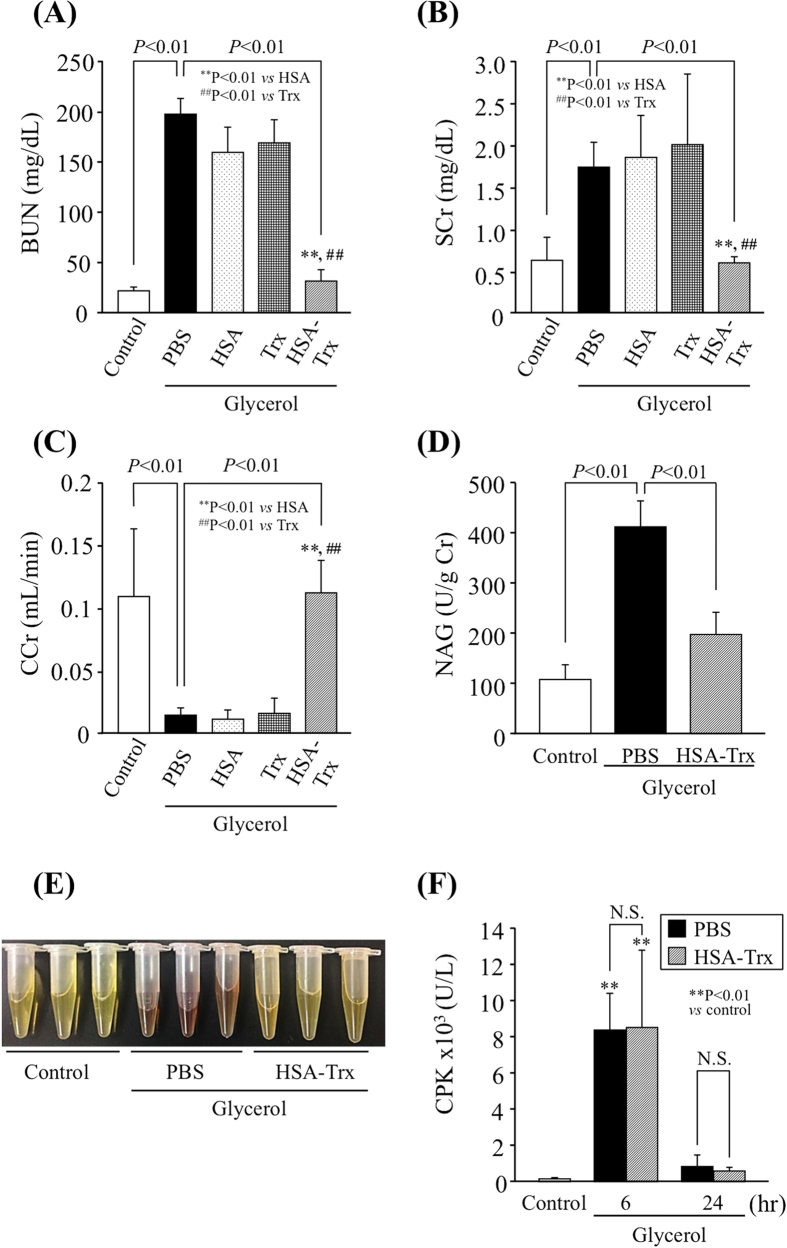
Renoprotective effect of HSA-Trx on glycerol-induced nephropathy. Changes in the levels of (**A**) blood urea nitrogen (BUN), (**B**) serum creatinin (Scr), (**C**) creatinine clearance (CCr), (**D**) urinary N-acetyl-β-D-glucosaminidase (NAG) activity and (**E**) urine-color at 24 hr after an intravenous injection of 10 mL/kg of 50% glycerol. PBS, HSA-Trx (400 nmol/kg), HSA (400 nmol/kg) or Trx (400 nmol/kg) was injected just before glycerol injection. (**F**) Changes of plasma creatine phosphokinase (CPK) at 6 and 24 hr after intravenous injection of 10 mL/kg of 50% glycerol treated with or without HSA-Trx. HSA-Trx had no effect on the intensity of the rhabdomyolysis. Each column represents the mean ± SD (n = 6). ^**^P < 0.01 *vs* HSA; ^##^P < 0.01 *vs* Trx. N.S., no significant change.

The experimental schedule is summarized in Fig. 1. Our preliminary study using a dose of 100, 200 or 400 nmol/kg of HSA-Trx clearly indicated that HSA-Trx had a renoprotective effect against a glycerol-induced AKI mouse and that the effect was dose-dependent (data not shown). The pre-administration of 400 nmol/kg HSA-Trx resulted in the nearly complete suppression in renal injury. Therefore, we used a dose of 400 nmol/kg HSA-Trx in the following study. In fact, the pre-administration of a single intravenous dose of HSA-Trx (400 nmol/kg) (Fig. 1A) significantly attenuated the increase in BUN levels (Fig. 2A), SCr (Fig. 2B), and the decrease in CCr (Fig. 2C) at 24 hr after glycerol administration in comparison with the phosphate buffered-saline (PBS) group. In contrast, the administration of an equimolar amount of HSA (400 nmol/kg) or Trx (400 nmol/kg) had no effect on improving glycerol-induced renal dysfunction in comparison with HSA-Trx. We also measured urinary N-acetyl-β-D-glucosaminidase (NAG) activity, a more specific biomarker for tubular injury^19^. As shown in Fig. 2D, urinary NAG activity caused by glycerol administration was significantly attenuated by the treatment of HSA-Trx. Under the same experimental conditions, the tea-colored urine that was observed in the glycerol injection group was normalized in the HSA-Trx treatment group (Fig. 2E). To rule out the possibility that HSA-Trx directly inhibited the rhabdomyolysis response to a glycerol injection, we assessed plasma creatine phosphorkinase (CPK) levels in both the PBS and HSA-Trx treated mice. As shown in Fig. 2F, a similar elevation in plasma CPK levels was observed in both PBS and HSA-Trx treated mice, indicating that HSA-Trx did not affect the rhabdomyolysis response.

### Effect of HSA-Trx on renal histological alterations caused by glycerol

**Figure 3:**
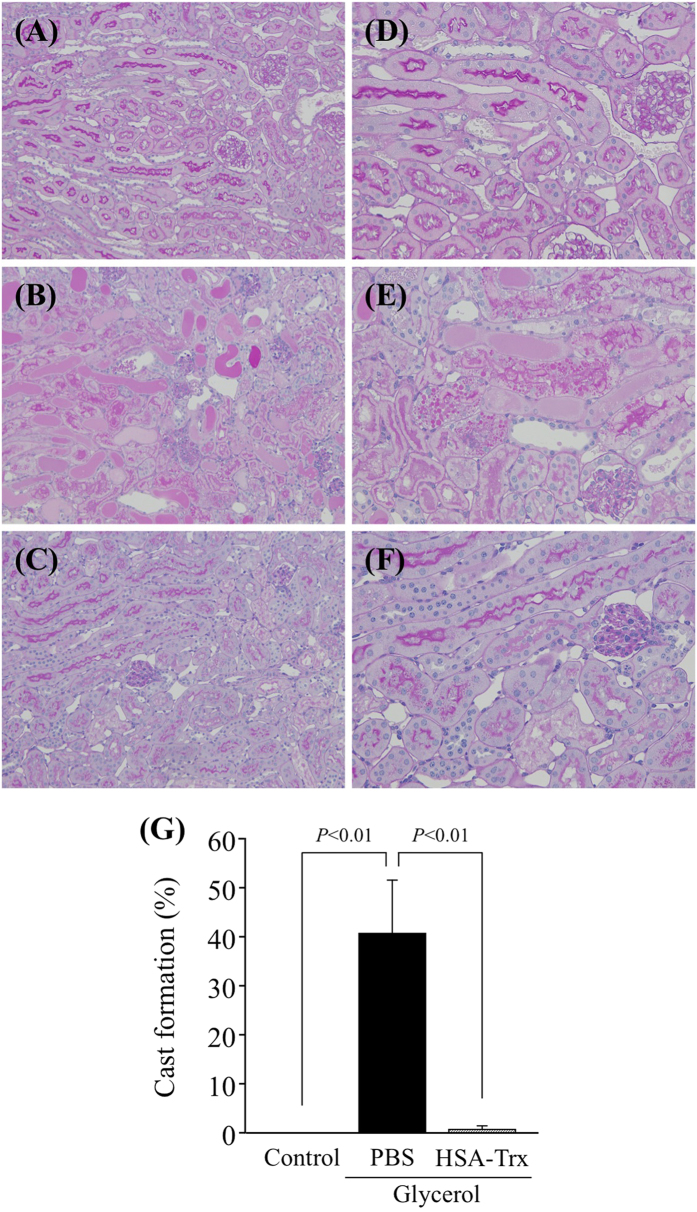
Histological assessment of the kidney of control or glycerol-induced nephropathy mice with or without HSA-Trx pretreatment. Representative photomicrographs of PAS-stained kidney sections (**A**–**F**) and a semiquantitative scoring analysis of cast formation are presented (**G**) at 24 h after intravenous injection of 10 mL/kg of 50% glycerol are presented. (A and D) Control mice, (**B**,**E**) mice with glycerol after a PBS injection and (**C**,**F**) mice with glycerol after a HSA-Trx injection. PBS or HSA-Trx was injected just before the 10 mL/kg of 50% glycerol injection. The marked tubular injuries and cast formation caused by glycerol were diminished as the result of the HSA-Trx treatment. Cast formation induced by glycerol was significantly suppressed as he result of the HSA–Trx treatment (**G**). For the semiquantitative analysis of morphological changes, 20 high- magnification (×200) fields of the cortex and the outer stripe of the outer medulla in mice were randomly selected. Original magnifications: ×200 (**A**–**C**); ×400 (**D**–**F**). Data are presented as mean ± SD (n = 4 ~ 5). Statistical analyses were performed using the Tukey multiple comparison.

Figure 3 shows histological alterations, as evidenced by periodic acid-Schiff (PAS) staining and a semiquantitative scoring analysis of kidneys of the control and glycerol groups with and without HSA-Trx. The glycerol injection group showed tubular damage, foamy and hyaline droplet degeneration of the tubular cells, in addition to cast formation compared with the control group (Fig. 3B,E). Specifically, the most severe and pronounced alterations were observed in the renal cortico-medullary boundary zone (the cortex and outer stripe of the outer medulla) rather than in the inner stripe of the outer medulla or inner medulla. However, such histological features were largely inhibited by the HSA-Trx treatment (Fig. 3C,F,G). These morphological changes are entirely consistent with the altered renal function as shown in Fig. 2.

### Effect of HSA-Trx on renal tubular apoptosis caused by glycerol

**Figure 4:**
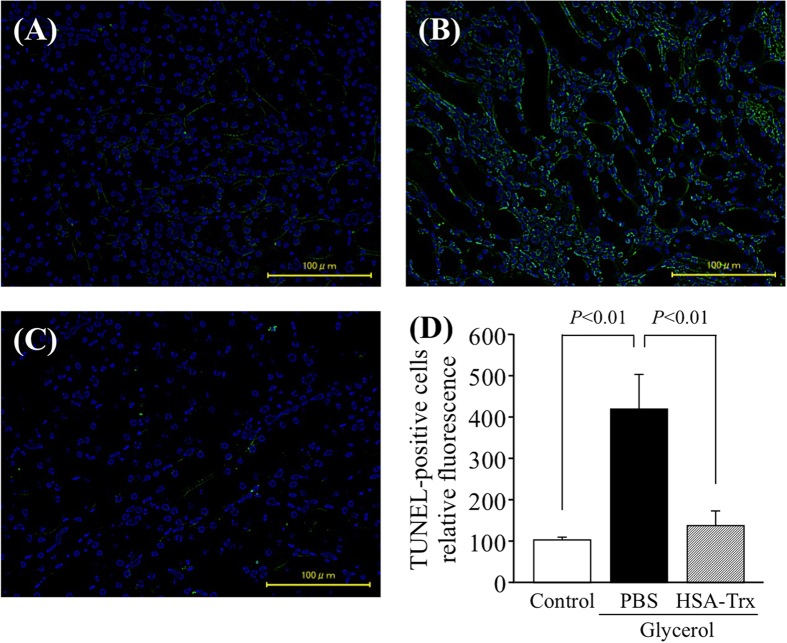
TUNEL staining of the kidneys of control or glycerol-induced nephropathy mice with or without HSA-Trx. Representative photomicrographs of TUNEL-stained (green) kidney sections (**A**–**C**) at 24 h after intravenous injection of 10 mL/kg of 50% glycerol are presented. (**A**) Control mice, (**B**) mice treated with glycerol after PBS injection, (**C**) mice treated with glycerol after HSA-Trx injection. Cells were also treated with DAPI (blue). (**D**) Image analysis of the extent and intensity of TUNEL-staining was performed. PBS or HSA-Trx was injected immediately prior to the injection of 10 mL/kg of a 50% glycerol solution. While the number of TUNEL-positive renal tubular cells were increased in the case of glycerol-induced nephropathy, the numbers were markedly decreased by HSA-Trx treatment. Data are presented as the mean ± SD (n = 4 ~ 5).

Terminal deoxynucleotidyl transferase-mediated deoxyuridine triphosphate nick-end labeling (TUNEL) immunostaining was performed to evaluate renal tubular apoptosis (Fig. 4). Kidneys from the glycerol group that had been administered PBS showed a marked increase in the number of TUNEL-positive tubular epithelial cells, mainly in the outer medulla (Fig. 4B,D). In contrast, the number of TUNEL-positive cells in the HSA-Trx group was markedly decreased compared with the group that had been treated with PBS (Fig. 4C,D).

### Effect of HSA-Trx on renal ROS induced by glycerol

**Figure 5:**
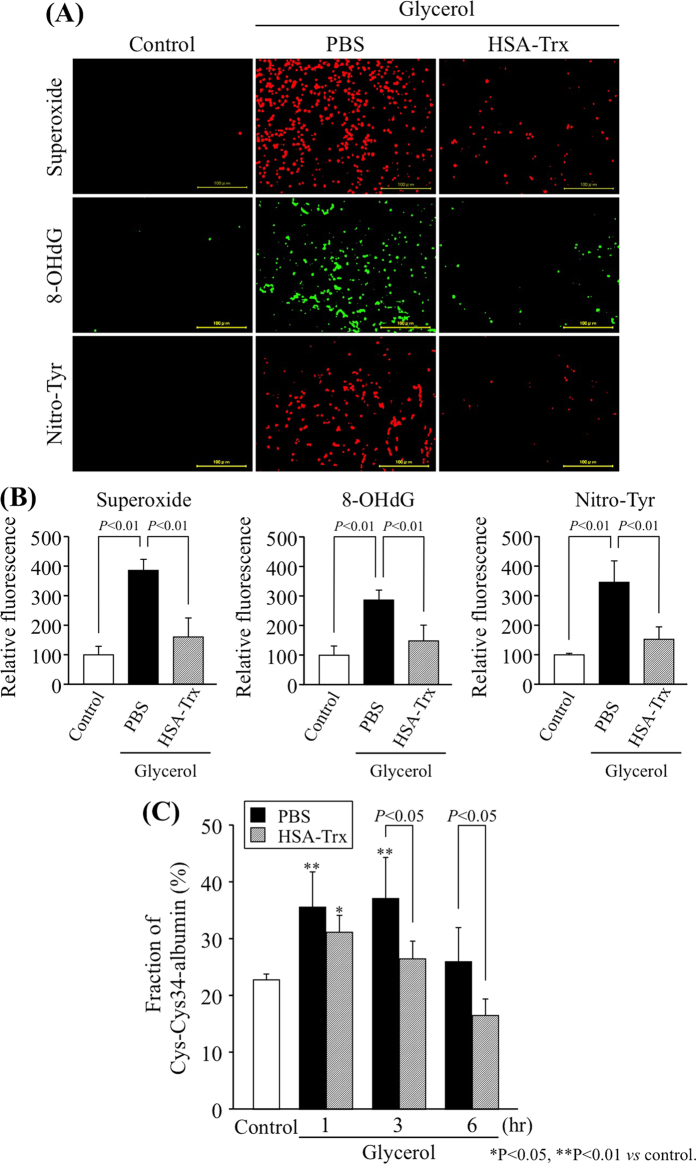
The redox effect of HSA-Trx on the renal and plasma oxidative stress in glycerol-induced nephropathy mice. (**A**) Representative photomicrographs of renal superoxide (DHE staining) (upper panels), immunostaining of renal 8-OHdG (8-hydroxy-2′-deoxygenase) (middle panels) and Nitro-Tyr (nitrotyrosine) (lower panels) at 24 h after the intravenous injection of 10 mL/kg of 50% glycerol in control mice, mice treated with glycerol after a PBS injection or after the injection of HSA-Trx. (**B**) Image analysis of the extent and intensity of staining was performed. (**C**) Plasma oxidative stress marker, fraction of Cys-Cys34-albumin, was measured by ESI-TOFMS at 1, 3 and 6 hr after glycerol treatment with or without HSA-Trx. HSA-Trx suppressed the glycerol-induced oxidative stress in kidney and plasma. Data are presented as mean ± SD (n = 4 ~ 5). ^*^P < 0.05, ^**^P < 0.01 *vs* control.

It has been demonstrated that heme released from myoglobin and hemeprotein, and free iron driven hydroxyl radicals are critical in the development of glycerol-induced nephropathy^8^,^9^. Therefore, to investigate whether HSA-Trx caused a decrease in ROS levels induced by glycerol, kidney sections at 24 h after glycerol administration were examined for superoxide production (dihydroethidium (DHE) staining: Fig. 5A) and immunostained to detect the presence of an oxidized derivative of deoxyguanosine (8-OHdG) (Fig. 5A). In addition, kidney sections were also immunostained for nitrotyrosine (Nitro-Tyr), a product produced by the reaction of reactive nitrogen species (RNS) and protein (Fig. 5A). In the glycerol group that had been administered PBS, the number of superoxide, 8-OHdG and Nitro-Tyr-positive cells in the renal tubules, mainly in the outer medulla, were markedly increased compared to the control group. In contrast, the HSA-Trx administration clearly resulted in a decrease in the number of superoxide, 8-OHdG and Nitro-Tyr-positive cells compared with the PBS treatment group (Fig. 5A,B). We also determined the effect of HSA-Trx on plasma oxidative stress using Cys34-cysteinylated albumin (Cys-Cys34-albumin), which was recently developed by us as a marker of plasma oxidative stress^24^,^25^,^26^. As shown in Fig. 5C, the level of Cys-Cys34-albumin in the plasma fraction was significantly increased at 1 hr and 3 hr after the glycerol injection, but was restored by the HSA-Trx treatment. These data suggest that HSA-Trx suppresses renal and plasma ROS levels in glycerol-treated mice.

### Effect of HSA-Trx on inflammatory cytokines and macrophage migration inhibitory factor levels in plasma induced by glycerol

**Figure 6:**
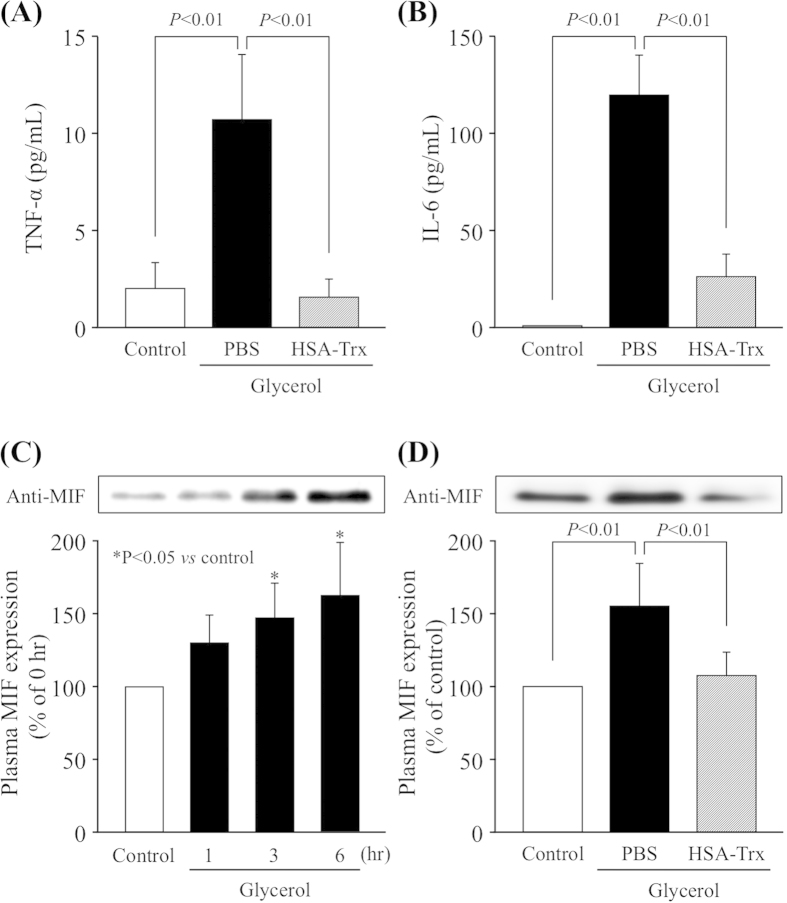
Effect of HSA-Trx on the expression of inflammatory cytokines and chemokines in glycerol-induced nephropathy mice. The expression of (**A**) TNF-α and (**B**) IL-6 in plasma was determined by ELISA kit at 24 h after 10 mL/kg of 50% glycerol injection. (**C**) MIF expression in plasma at 1, 3 and 6 hr after glycerol injection was analyzed by Western blotting. (**D**) The expression of MIF in plasma at 6 hr after glycerol injection. HSA-Trx suppressed the glycerol-induced TNF-α, IL-6 and MIF expression in plasma. Data are presented as the mean ± SD (n = 6). ^*^P < 0.05 *vs* control.

Glycerol-induced AKI causes an increase in the levels of a variety of inflammatory cytokines. Here, we examined the effect of HSA-Trx on TNF-α, IL-6 and MIF in plasma of glycerol-induced AKI mouse. As shown in Fig. 6A,B, the levels of TNF-α and IL-6 in plasma at 24 hr after the glycerol injection were significantly decreased to the control level as the result of the HSA-Trx treatment. Since there is no information regarding the changes in plasma MIF levels in rhabdomyolysis-associated AKI, the time profile of the plasma MIF level was monitored after the glycerol injection. As shown in Fig. 6C, the plasma MIF level was gradually increased for 6 hr after the glycerol injection. Intriguingly, the HSA-Trx treatment significantly suppressed plasma MIF levels at 6 hr after the glycerol injection (Fig. 6D). These data suggest that HSA-Trx exerts an anti-inflammatory action partially *via* suppressing MIF against glycerol-induced kidney damage.

### Effect of HSA-Trx on cellular ROS levels and cell viability induced by myoglobin in LLC-PK1 cells

**Figure 7:**
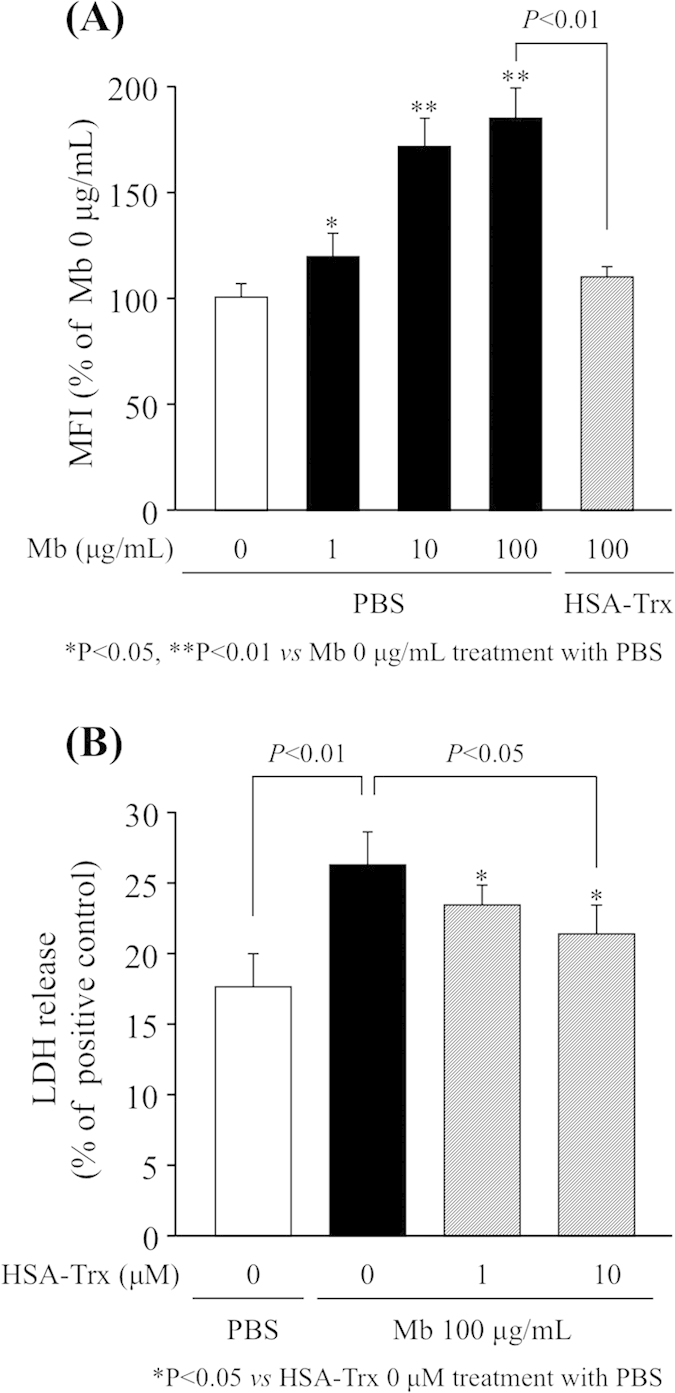
Effect of HSA-Trx on cellular ROS levels and cell viability induced by myoglobin in LLC-PK1 cell. (**A**) LLC-PK1 cells were incubated in 96-well plates (1 × 10^4^ cells/well) in Medium 199 containing 10% FBS at 37 °C for 24 h, and then incubated with 5 μM CM-H_2_DCFCA for 30 min in D-PBS. After removing the D-PBS from the wells, the cells were incubated with 10 μM HSA-Trx for 1 h, and then incubated with 0–100 μg/mL myoglobin for 3 h. Fluorescence intensity was measured at an excitation wavelength of 485 nm and an emission wavelength of 535 nm. Each column represents the mean ± SD (n = 4). ^*^P < 0.05, ^**^P < 0.01 *vs* 0 μg/mL myoglobin (Mb) treatment with PBS. (**B**) Cell viability induced by myoglobin was evaluated by measuring the release of LDH into the medium. LLC-PK1 cells were incubated in 24-well plates (5 × 10^4^ cells/well) in Medium 199 containing 10% FBS at 37 °C for 24 h. The cells were incubated with 1 or 10 μM HSA-Trx for 1 h, and then incubated with 100 μg/mL myoglobin for 4 h. The amount of LDH released into the medium from the LLC-PK1 cells was then measured. ^*^P < 0.05 *vs* 0 μM HSA-Trx treatment with PBS.

The effect of HSA-Trx on cellular ROS levels and cell viability (LDH release) induced by myoglobin was examined using porcine kidney epithelial cells (LLC-PK1 cells). The presence of myoglobin caused a significant increase in ROS production in a time-dependent manner, reaching a maximum at 100 μg/mL. Intriguingly, the extent of myoglobin-induced ROS levels were significantly suppressed in the presence of HSA-Trx (Fig. 7A). HSA-Trx also significantly suppressed the release of LDH from LLC-PK1 cells induced by myoglobin (Fig. 7B).

### Effect of HSA-Trx on the survival of lethal glycerol treated mice

**Figure 8:**
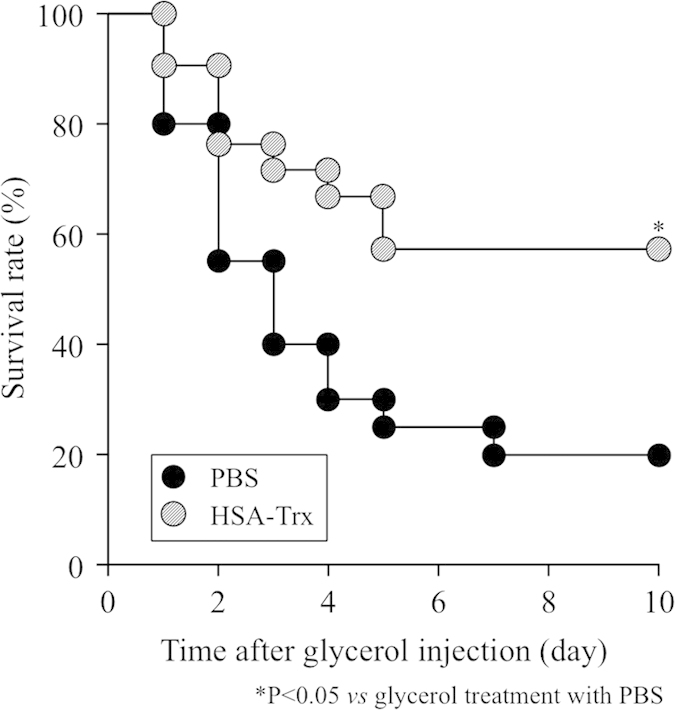
Effect of HSA-Trx on survival of lethal glycerol-treated mice. HSA-Trx (400 nmol/kg) was administered intraveniously just before 13 mL/kg of 50% glycerol injection. ^*^P < 0.05 vs glycerol treatment with PBS.

We investigated the effect of HSA-Trx on the survival of glycerol-induced AKI mice. The mice were challenged with a lethal dose of glycerol (13 mL/kg) just after the administration (iv) of PBS or HSA-Trx. As shown in Fig. 8, 20% of the PBS treated group mice were alive at 10 days after the glycerol injection. On the other hand, HSA-Trx administration significantly increased the survival rate, and 57% of the mice were still alive.

### Effect of the post-administration of HSA-Trx on glycerol treated mice

**Figure 9:**
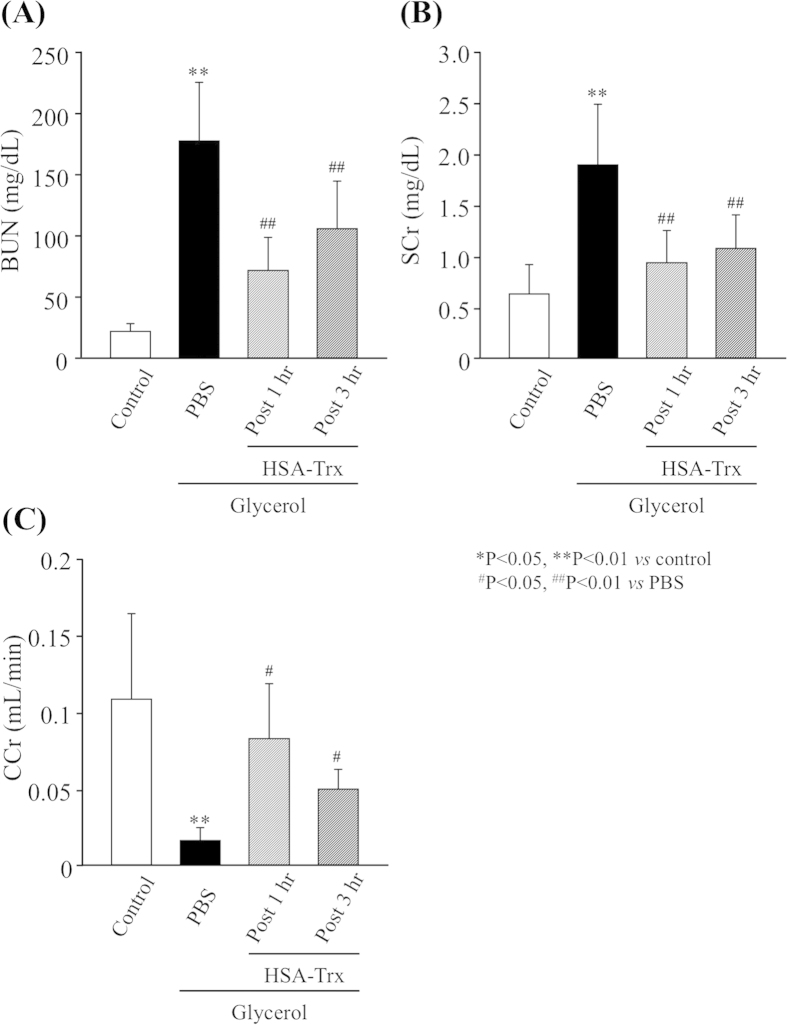
Effect of post-administration of HSA-Trx on glycerol-treated mice. HSA-Trx (400 nmol/kg) was intravenously administered 1 or 3 h after the injection of 10 mL/kg of a 50% glycerol solution. Changes in the levels of (**A**) blood urea nitrogen (BUN), (**B**) serum creatinin (Scr) and (**C**) creatinine clearance (CCr) at 24 h after intravenous injection of 10 mL/kg of 50% glycerol. Each column represents the mean ± SD (n = 6). ^*^P < 0.05, ^**^P < 0.01 *vs* control; ^#^P < 0.05, ^##^P < 0.01 *vs* PBS.

To investigate the potential of HSA-Trx as a rescue therapy after glycerol treatment, it was administered 1 hr and 3 hr following a glycerol injection. As shown in Fig. 9, the administration of HSA-Trx at 1 hr and 3 hr after the glycerol injection significantly inhibited the elevation in BUN and SCr levels or the reduction of CCr, respectively. These data indicate that the post-administration of HSA-Trx could also ameliorate a renal injury in glycerol-treated mice.

## Discussion

AKI associated with myoglobinuria is the most serious complication of rhabdomyolysis. Although it may be life-threatening, interventions, such as the use of mannnitol and bicarbonate, to preserve renal function has little clinical evidence. Therefore, a new treatment strategy that could mitigate rhabdomyolysis-induced AKI would be highly desirable.

We recently produced an HSA-Trx fusion protein that has long acting anti-oxidative activity and has superior preventing effects to NAC, a typical anti-oxidant, in drug-induced AKI, such as experimental contrast-induced nephropathy or cisplatin-induced nephropahy mouse models^19^,^20^. In the latter study, from a pharmacokinetic approach using FITC-labeled HSA-Trx, we demonstrated that HSA-Trx exerted its biological effect predominantly in the extracellular compartment, and partially in the intracellular compartment of tubular cells^20^. In addition, the pre- and post-administration of HSA-Trx also had suppressive effects against bleomycin-induced pulmonary fibrosis^18^ and acetaminophen-induced hepatitis^21^ by virtue of its ability to modulate oxidative stress and MIF. Therefore, it would be expected that HSA-Trx has the potential for use in the treatment or prevention of rhabdomyolysis-associated AKI.

Although the exact mechanisms by which rhabdomyolysis impairs the glomerular filtration rate are currently unclear, the available experimental evidence suggests that oxidative stress^7^, inflammation^11^ and tubular apoptosis^7^ all play a role. Several previous studies reported that the administration of N-acetylcystein (NAC) prevented renal dysfunction in a myoglobinuric AKI model^7^,^27^. However, the renoprotective effect of NAC is controversial. Although Kim J *et al.* demonstrated that the renoprotective effect of NAC in glycerol-induced AKI, NAC (150 mg/kg) provided only partial protection against AKI in rats^7^. These data suggest that an anti-oxidative agent with a short half-life is not an ideal compound for use in the treatment of glycerol-induced AKI. HSA-Trx markedly attenuated glycerol-induced renal and plasma oxidative stress, as evidenced by an evaluation of renal superoxide production, the accumulation of 8-OHdG and Nitro-Tyr in addition to the plasma fraction of Cys-Cys34-albumin (Fig. 5). Trx suppresses oxidative stress *via* a number of mechanisms. For instance, Trx directly scavenges ROS such as hydroxyl radicals via the reversible oxidation of its redox-active cysteine residues^28^. This mechanism likely contributes to the anti-oxidative effect of HSA-Trx against glycerol-induced renal injury. It is well known that HSA also plays an important role in the anti-oxidant defense system in the blood circulation^29^. Therefore, it is highly possible that the HSA part of HSA-Trx also contributes to the anti-oxidative activity of the molecule. However, as shown in Fig. 2, the administration of an equal molar amount of HSA or Trx alone had no renoprotective effect on glycerol-treated mice in comparison with the HSA-Trx treatment group. These findings indicate that the anti-oxidative effect of HSA-Trx is mainly dependent on the extended action of Trx that results from its fusion with HSA.

It has been documented that a series of inflammatory changes occur in glycerol-induced AKI. Proinflammatory cytokine, TNF-α, has also been shown to be increased after an injection of glycerol. In particular, TNF-α is thought to play a central role in mediating renal injuries *via* the induction of apoptosis and ROS production, subsequently coordinating the activation of a large network of chemokines and cytokines in the kidney^30^. In fact, Shulman L *et al.* reported that glycerol-inuced AKI can be partially ameliorated by the pre-administration of an anti-TNF-α antibody^12^. Trx has been reported to exert its anti-inflammatory properties by suppressing the migration and/or infiltration and extravascular leakage of leukocytes *via* modulating the expression of MIF^31^,^32^, resulting in a reduced expression of TNF-α and IL-6^33^. MIF is induced in a broad range of oxidative stress conditions and the resulting inflammation that develops as a result of certain types of pathological conditions such as sepsis, rheumatoid arthritis, inflammatory bowel disease, hepatitis and pulmonary disease^34^. In such cases, MIF promotes the production of TNF-α, IL-6 and other cytokines, as well as the production of ROS, such as nitric oxide and superoxide anions^14^. The findings reported herein show, for the first time, that plasma MIF levels were markedly increased prior to the elevation of inflammatory cytokines such as TNF-α and IL-6 under conditions of glycerol-induced AKI as similar to other inflammatory diseases, and HSA-Trx markedly suppressed the glycerol-induced elevation of plasma MIF as well as TNF-α and IL-6 (Fig. 6). Tamaki H *et al.* previously reported that exogenous Trx suppresses the production and release of MIF in a human monocyte cell line, suggesting Trx and MIF counteract each other during the inflammatory process^32^. Therefore, suppressing the effect of exogenous HSA-Trx on MIF expression could have occurred by the same mechanism as Trx. However, the mechanism underlying the association between Trx and MIF is not well understood at present. In this study, the modulation of MIF expression by HSA-Trx is likely to be a crucial factor for the inhibition of cytokine production. Further studies using MIF knockout mice might be necessary to clarify the contribution of MIF in this model.

Since rhabdomyolysis-associated AKI occurs as the result of a variety of situations such as accidents and hospitalization etc, and the outcome of this disease depends on the extent of kidney damage, therapeutics with superior efficacy that could be applied to both prevention and treatment is highly desirable. The findings of this study indicate that HSA-Trx is not only able to prevent glycerol-induced AKI by pre-administration, but also represents an effective by post-administration. It therefore appears the HSA-Trx has a potential as a promising and versatile resue therapy against rhabdomyolysis-asscociated AKI.

## Conclusions

Based on the results of this study, we propose that the pre- and post-administration of HSA-Trx could be therapeutically beneficial in the treatment of rhabdomyolysis-induced AKI as a consequense of its long-lasting anti-oxidative, anti-inflammatory and anti-apoptotic action.

## Methods

### Materials

The Pichia Expression Kit was purchased from Invitrogen (Carlsbad, CA, USA). Glycerol and other chemicals were of reagent grade or of the highest purity available commercially. All methods were carried out in accordance with approved guidelines. All experimental protocols were approved by Kumamoto University.

### Production of HSA-Trx fusion protein

The genetic fusion of Trx and HSA was performed and the fusion protein was produced following a previously reported method^16^,^17^,^18^,^19^,^20^,^21^. Briefly, transformed Pichia pastoris cells were incubated in 5 L of BMGY liquid media (1% yeast extract, 2% peptone, 100 mM potassium phosphate (pH 6.0), 1.34% yeast nitrogen base with ammonium sulfate without amino acids, 4 × 10 − 5% biotin, 1% glycerol) (growth phase) for 2 days (OD600 = 2), and then cultured in 800 mL of BMMY media that contained a protein expression inducer as well as a carbon source, methanol (1% yeast extract, 2% pepton, 100 mM potassium phosphate (pH 6.0), 1.34% yeast nitrogen base with ammonium sulfate without amino acids, 4 × 10 − 5% biotin, 1% methanol) (protein induction phase) for 3 days at 30 °C. Methanol was added daily, to permit the concentration of methanol to be maintained at a level of 1% in order to sustain the protein expression induction effect. Purification of the fusion protein was initially carried out by chromatography on a Blue Sepharose 6 Fast Flow column (GE Healthcare, Tokyo, Japan) equilibrated with 200 mM sodium acetate buffer (pH 5.5) after dialysis against the same buffer. Next, using AKTA prime, a 5 mL HiTrap Phenyl HP column (GE Healthcare, Tokyo, Japan) was used for hydrophobic chromatography, under the following conditions: Buffer A, 50 mM Tris–HCl + 1.5 M ammonium sulfate, pH 7.0; Buffer B, 50 mM Tris–HCl, pH 7.0; Gradient, 0–100% (Buffer B) 100 mL; Flow rate, 3 mL/min. The desired fusion protein was analyzed by SDS-PAGE using a 15% polyacrylamide gel, with Coomassie blue R250 staining. The fusion protein was estimated to be more than 95% pure.

### Mouse model of glycerol-induced AKI

All animal experiments were performed according to the guidelines, principles, and procedures for the care and use of laboratory animals of Kumamoto University. ICR mice (male, 6 weeks, Kyudo Co, Kumamoto, Japan) were maintained in a temperature-controlled room with a 12-hr dark/light cycle and ad libitum access to food and water. To induce rhabdomyolysis, the two hind limbs of the animals were intramuscularly injected with 10 mL/kg of 50% glycerol or were injected with saline as a control. Water was withheld for 24 h before the glycerol injection to increase the incidence of the renal failure. The mice were randomized to receive the HSA-Trx (400 nmol/kg) or phosphate-buffered saline (PBS) at the same time of the glycerol injection. PBS or HSA-Trx were injected via the tail vein. The mice were allowed to recover in metabolic cages for an additional 24 h, and urine samples were collected and used for the determination urinary creatinine excretion and N-acetyl-β-D-glucosaminidase (NAG) activity. After collecting blood samples for the determination of BUN, SCr, inflammatory cytokines and chemokines, the mice were sacrificed under diethyl ether anesthesia at 24 h after the injection of glycerol. The kidneys were removed and bisected in the equatorial plane, the right kidney was homogenized for the Western blotting analysis, and the left kidney was fixed in phosphate buffered 4% formalin and prepared for routine histological examination.

### Biochemical evaluation of blood and urine samples

Mean SCr and urinary creatinine concentrations were measured by enzymatic methods, using the respective assay kits (Wako Pure Chemical, Osaka, Japan). The mean BUN concentration was determined by the diacetylmonoxime method using an assay kit (Wako Pure Chemical). The activity of NAG in urine was determined by the enzymatic degradation of the substrate sodium cresol sulfonephtha-leinyl N-acetyl-β-D-glucosaminide using a commercial assay kit (Shionogi Pharmaceutical, Osaka, Japan). NAG activity was expressed as units per gram of urinary creatinine. Creatinine clearance during 24 h after the injection of glycerol was calculated as ml/min.

### Histologic examination of renal tissues

The left kidney, fixed in 10% phosphate buffered formalin, was dehydrated in a graded series of ethyl alcohol concentrations and embedded in paraffin. Kidney blocks were cut into 2-μm sections and then subjected to periodic acid-Schiff (PAS) staining for morphologic analysis and TUNEL staining for cell apoptosis and immunohistochemistry (8-hydroxy-2-deoxygenase (8-OHdG) and nitrotyrosine (Nitro-Tyr)). PAS-stained tissue sections were viewed by light microscopy at magnifications of ×200 or ×400. For semiquantitative analysis of morphological changes, 20 high-magnification (×200) fields of the cortex and outer stripe of the outer medulla were randomly selected. All quantifications were performed in a blinded manner. For TUNEL staining, the sections were stained using an *In situ* cell death detection kit, Fluorescein (Roche, Basel, Switzerland) according to the manufacturer's protocol for paraffin-embedded sections. Cells were also treated with DAPI (Dojin Chemical, Kumamoto, Japan). For the immunohistochemistry of 8-OHdG and Nitro-Tyr, first, antigen retrieval was conducted by means of immunosaver (Nisshin EM Corporation, Tokyo, Japan). A solution containing 50 mM Tris–HCl + 0.1% Tween-20 (T-TB) was then used to solubilize the kidney slices, followed by blocking with Block Ace (Dainippon Pharmaceutics, Osaka, Japan) at room temperature for 15 min. The primary antibody reaction was then conducted overnight at a temperature below 4 °C. In addition, the primary antibody containing inducible 8-OHdG [15A3] (Santa Cruz, California, USA, cat#: sc-651) or Nitro-Tyr (Santa Cruz California, USA, cat#: sc-66036) was diluted 50 times prior to use. The kidney slices were then washed with T-TB, followed by reaction with the secondary antibody at room temperature for 1.5 h. For 8-OHdG immunostaining, Alexa Fluor 488 goat anti-mouse IgG (H + L) (Invitrogen, Tokyo, Japan), for Nitro-Tyr immunostaining, Alexa Fluor 546 goat anti-mouse IgG (H + L) (Invitrogen, Tokyo, Japan) was diluted 100 times before use. After the reaction, the slide was observed using a Microscope (Keyence, BZ-8000, Osaka, Japan).

### Measurement of renal superoxide

Dihydroethidium (DHE) was used to evaluate renal superoxide concentrations *in situ*, as described in detail elsewhere^35^. After the reaction, the slide was observed using a Microscope (Keyence, BZ-8000, Osaka, Japan). DHE fluorescence of renal sections was quantified using the Image J analysis software. The mean fluorescence was quantified and expressed relative to values obtained in control rats.

### Measurement of Cys-Cys34-albumin by ESI-TOFMS

This measurement was performed according to our previous methods^25^,^26^. The collected blood was immediately diluted (×10) with 0.5 M sodium citrate buffer (pH 4.3) to stabilize the albumin in the reduced form. Each plasma fraction was separated by centrifugation (4 °C for 15 min, 2000 g). After collection, plasma samples were stored at −80 °C until used in batch analyses. A 5 μL aliquot of plasma was added to 495 μL of 50 mM sodium phosphate buffer (pH 6.0). A solid phase extraction (SPE) column (Bond Elute-C18 EWP 200 mg/3cc, Varian, Inc., CA) was initialized with 10/90 water/acetonitrile containing 0.1% formic acid, and equilibration was then performed with water (1 mL). The above-mentioned diluted plasma sample was applied to the equilibrated SPE column. The column was washed with 10% acetonitrile (2 mL) containing 0.1% formic acid, and albumin was eluted with 90% acetonitrile containing 0.1% formic acid. A 2 μL aliquot of the eluent was flow injected into the ESI-TOFMS (microTOF^®^; Bruker Daltonics Inc. USA) at a flow rate of 15 μL/min with 10/90 water/acetonitrile containing 0.1% formic acid using the auto sampler of Ultimate 3000 (Dionex, Idstein, Germany). The data were acquired by the MicroTOF^®^ software (Bruker-Daltonics) and processed for Maxent deconvolution using DataAnalysis^®^ software (Bruker-Daltonics). The deconvolution mass range was set to be from 66000 to 68000 Da. And the mass peak of albumin and its modified molecules such as Cys-Cys34-albumin or glycated albumin, was automatically assigned and converted to an output text file using script with a resolving power of 10000 m/dm and an absolute intensity threshold of 1000. The fraction of Cys-Cys34-albumin (%) was calculated by (Cys-Cys34-albumin/(Cys-Cys34-albumin + reduced albumin)) × 100.

### Quantification of plasma TNF-α and IL-6 level

TNF-α and IL-6 ELISA kit were purchased from Biolegend (San Diego, CA). The amounts of TNF-α and IL-6 in plasma at 24 hr after glycerol administration were measured according to the manufacturer's protocol.

### Western blot analysis of plasma MIF expression

Western blotting of MIF chemokine in plasma at 0, 1, 3 and 6 hr after glycerol administration was performed using the following protocol. Each plasma sample was separated by 12.5% SDS-PAGE and transferred onto polyvinylidene difluoride membranes (Immobilon-P; Millipore, Bedford, MA) by wet electroblotting. The membranes were blocked for 1 hour at room temperature with 5% skim milk in PBS. The membranes were washed three times with PBS containing 0.05% Tween 20 (PBS-T) and incubated for 2 hours at room temperature with a 400 ng/ml primary mouse monoclonal antibody against the N terminus of MIF of human origin, which has cross-reactivity with mouse MIF (sc-271631; Santa Cruz Biotechnology Inc., Santa Cruz, CA) in PBS-T. The membranes were washed 3 times with PBS-T and incubated with the secondary antibody (horseradish peroxidase-linked anti-mouse IgG [H1L]; Invitrogen, Carlsbad, CA) for 1.5 hours at room temperature. The membranes were washed 3 times with PBS-T, and immunoblots were visualized using the SuperSignal West Pico chemiluminescent substrate (Pierce Biotechnology Inc.) with LAS-4000EPUVmini (Fujifilm, Tokyo, Japan).

### Survival analysis

For the analysis of the survival curve, The mice was challenged with a lethal dose of glycerol (13 mL/kg) just after the administration (iv) of PBS or HSA-Trx, and were then monitored for 10 days.

### Cell cultures

LLC-PK1 cells were used as a model of porcine kidney epithelial cells. LLC-PK1 cells (ATCC, Manassas, VA) was cultured at 37 °C in 5% CO_2_ in Medium 199 containing 10% FBS.

### Measurement of intracellular ROS levels and cell viability

To measure ROS levels (mostly peroxide), CM-H_2_DCFDA, a ROS-sensitive fluorescent dye, was used as a ROS probe. To evaluate the effect of HSA-Trx on myoglobin induced ROS production, LLC-PK1 cells were incubated in 96-well plates (1 × 10^4^ cells/well) in Medium 199 containing 10% FBS at 37 °C for 24 h, and then incubated with 5 μM CM-H_2_DCFDA for 30 min in D-PBS. After removing the D-PBS from the wells, the LLC-PK1 cells were incubated with 10 μM HSA-Trx for 1 h, and then incubated with 0–100 μg/mL myoglobin for 3 h. Fluorescence intensity was measured at an excitation wavelength of 485 nm and an emission wavelength of 535 nm using a fluorescence microplate reader (SPECTRA FLUOR, TECAN, Mannedorf, Switzerland). To measure the release of LDH (Wako Chemicals, Tokyo, Japan) into the medium, LLC-PK1 cells were incubated in 24-well plates (5 × 10^4^ cells/well) in Medium 199 containing 10% FBS at 37 °C for 24 h. The cells were incubated with 1 or 10 μM HSA-Trx for 1 h, and then incubated with 100 μg/mL myoglobin for 4 h. The amount of LDH released into the medium from the LLC-PK1 cells was then measured.

### Statistical analyses

The means for groups were compared by analysis of variance followed by Tukey's multiple comparison. The survival rates were compared using Kaplan-Meier survival curves and the log-rank test. A probability value of *P* < 0.05 was considered to be significant.

## Additional Information

**How to cite this article**: Nishida, K. *et al.* Renoprotective effect of long acting thioredoxin by modulating oxidative stress and macrophage migration inhibitory factor against rhabdomyolysis-associated acute kidney injury. *Sci. Rep.* **5**, 14471; doi: 10.1038/srep14471 (2015).

## Supplementary Material

Supplementary Information
