# Supplementary material for: Renoprotective effect of long acting thioredoxin by modulating oxidative stress and macrophage migration inhibitory factor against rhabdomyolysis-associated acute kidney injury
Source: Sci Rep. 2015 Sep 28;5:14471. doi: 10.1038/srep14471 (PMC4585989; doi:10.1038/srep14471)
Supplement: Supplementary Information [file srep14471-s1.pdf]

Supplemental Material

Accompanying the manuscript

Renoprotective effect of long acting thioredoxin by modulating oxidative stress and macrophage migration inhibitory factor against rhabdomyolysis-associated acute kidney injury

Kento Nishida, Hiroshi Watanabe, Shigeru Ogaki, Azusa Kodama, Ryota Tanaka, Tadashi Imafuku, Yu Ishima, Victor Tuan Giam Chuang, Masao Toyoda, Masumi Kondoh, Qiong Wu , Masafumi Fukagawa, Masaki Otagiri, and Toru Maruyama

This PDF file includes:

Figs. S1 and S2

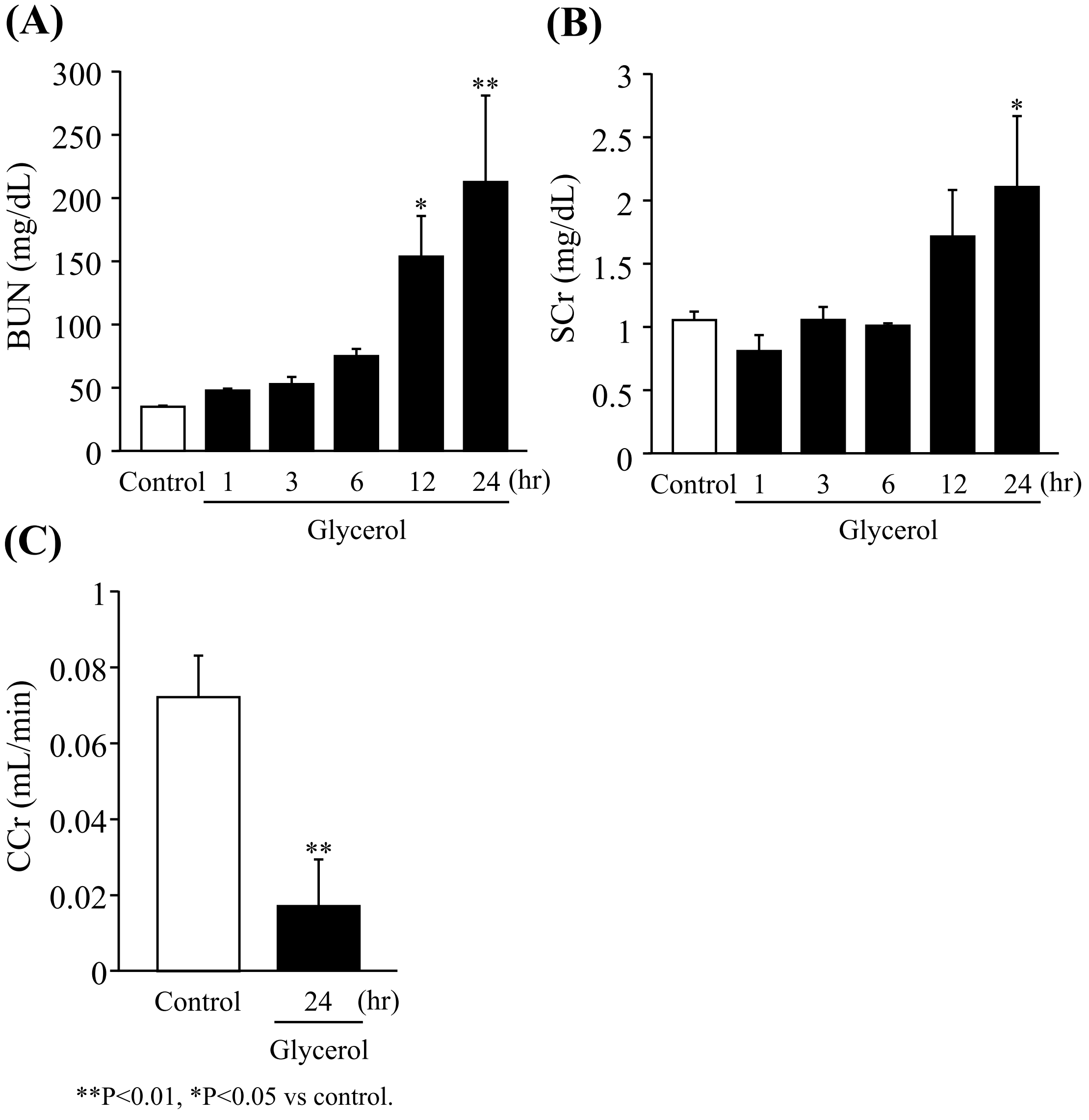

Figure S1. Changes in the levels of (A) blood urea nitrogen (BUN), (B) serum creatinin (Scr), (C) creatinine clearance (CCr) after an intravenous injection of glycerol. The mouse model of rhabdomyolysis-associated AKI, induced by the administration of 8 mL/kg of a 50% glycerol solution, showed elevations in BUN and SCr in a time dependent manner until 24 hr after glycerol administration. Decreased in CCr were observed at 24 hr after glycerol administration. *P<0.05,**P<0.01 *vs* control.

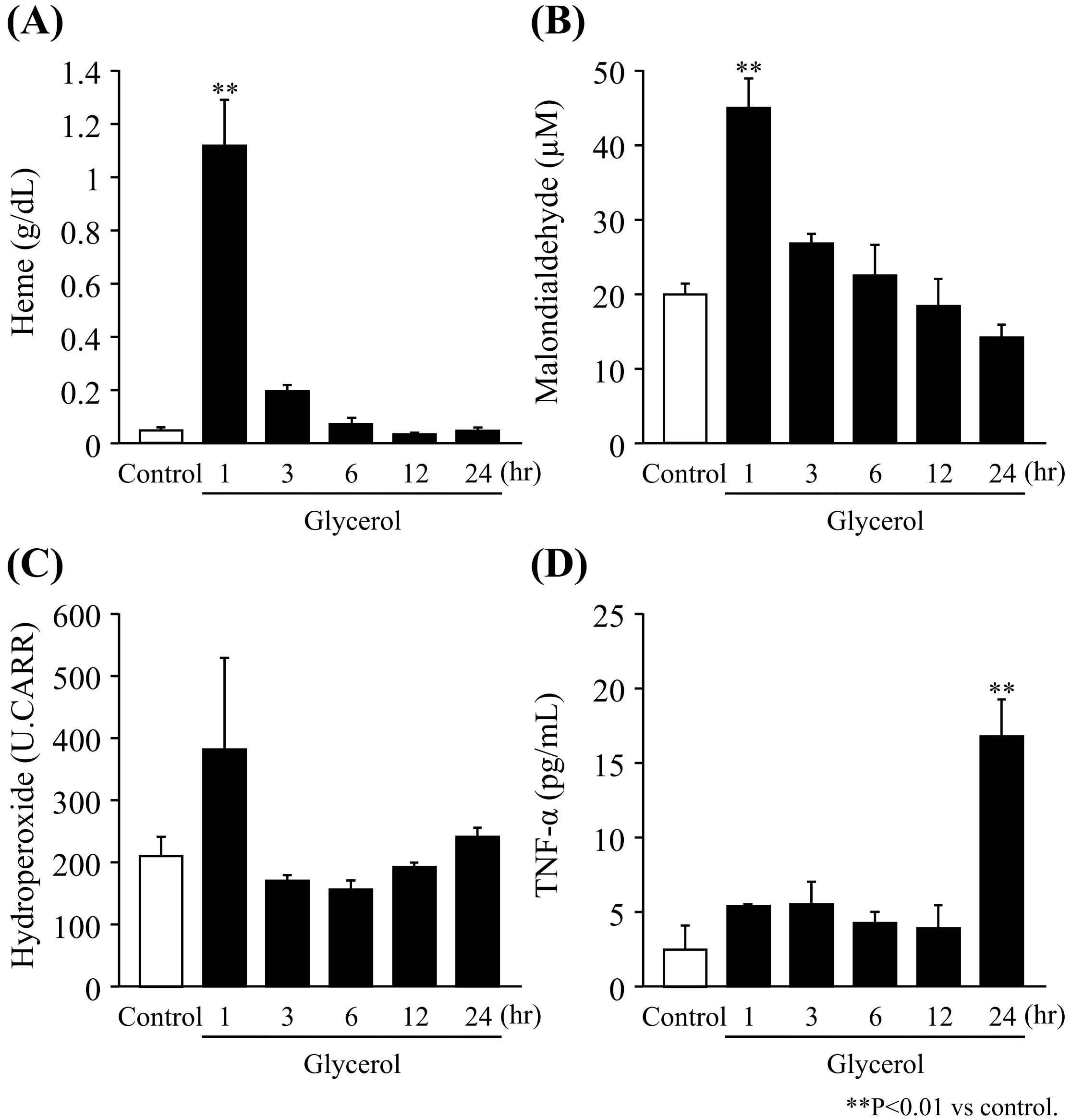

Figure S2. Plasma levels of (A) heme, (B) malondialdhyde, (C) hydroperoxide and (D) TNF-α after an intravenous injection of glycerol.

Levels of plasma oxidative stress markers such as heme, malondialdehyde and hydroperoxide reached a maximum at 1 h, and proinflammatory cytokine TNF-α was increased at 24 h after glycerol administration. **P<0.01 *vs* control.
